# Comparison of the Physical Activity Measured by a Consumer Wearable Activity Tracker and That Measured by Self-Report: Cross-Sectional Analysis of the Health eHeart Study

**DOI:** 10.2196/22090

**Published:** 2020-12-29

**Authors:** Alexander J Beagle, Geoffrey H Tison, Kirstin Aschbacher, Jeffrey E Olgin, Gregory M Marcus, Mark J Pletcher

**Affiliations:** 1 Department of Medicine University of California San Francisco San Francisco, CA United States; 2 Division of Cardiology Department of Medicine University of California San Francisco San Francisco, CA United States; 3 Department of Epidemiology and Biostatistics University of California San Francisco San Francisco, CA United States

**Keywords:** exercise, body mass index, overweight, obesity, fitness trackers, self-report, adult, mHealth, public health, cardiovascular diseases

## Abstract

**Background:**

Commercially acquired wearable activity trackers such as the Fitbit provide objective, accurate measurements of physically active time and step counts, but it is unclear whether these measurements are more clinically meaningful than self-reported physical activity.

**Objective:**

The aim of this study was to compare self-reported physical activity to Fitbit-measured step counts and then determine which is a stronger predictor of BMI by using data collected over the same period reflecting comparable physical activities.

**Methods:**

We performed a cross-sectional analysis of data collected by the Health eHeart Study, a large mobile health study of cardiovascular health and disease. Adults who linked commercially acquired Fitbits used in free-living conditions with the Health eHeart Study and completed an International Physical Activity Questionnaire (IPAQ) between 2013 and 2019 were enrolled (N=1498). Fitbit step counts were used to quantify time by activity intensity in a manner comparable to the IPAQ classifications of total active time and time spent being sedentary, walking, or doing moderate activities or vigorous activities. Fitbit steps per day were computed as a measure of the overall activity for exploratory comparisons with IPAQ-measured overall activity (metabolic equivalent of task [MET]-h/wk). Measurements of physical activity were directly compared by Spearman rank correlation. Strengths of associations with BMI for Fitbit versus IPAQ measurements were compared using multivariable robust regression in the subset of participants with BMI and covariates measured.

**Results:**

Correlations between synchronous paired measurements from Fitbits and the IPAQ ranged in strength from weak to moderate (0.09-0.48). In the subset with BMI and covariates measured (n=586), Fitbit-derived predictors were generally stronger predictors of BMI than self-reported predictors. For example, an additional hour of Fitbit-measured vigorous activity per week was associated with nearly a full point reduction in BMI (–0.84 kg/m^2^, 95% CI –1.35 to –0.32) in adjusted analyses, whereas the association between self-reported vigorous activity measured by IPAQ and BMI was substantially smaller in magnitude (–0.17 kg/m^2^, 95% CI –0.34 to –0.00; *P*<.001 versus Fitbit) and was dominated by the Fitbit-derived predictor when compared head-to-head in a single adjusted multivariable model. Similar patterns of associations with BMI, with Fitbit dominating self-report, were seen for moderate activity and total active time and in comparisons between overall Fitbit steps per day and IPAQ MET-h/wk on standardized scales.

**Conclusions:**

Fitbit-measured physical activity was more strongly associated with BMI than self-reported physical activity, particularly for moderate activity, vigorous activity, and summary measures of total activity. Consumer-marketed wearable activity trackers such as the Fitbit may be useful for measuring health-relevant physical activity in clinical practice and research.

## Introduction

The prevalence of overweight and obesity has steadily increased worldwide since 1980 [1], with concomitant increases in all-cause mortality [2] and morbidity from diseases such as cardiovascular disease [3,4]. Increased physical activity in adults is associated with reductions in BMI, cardiovascular disease, and all-cause mortality [5-8], thereby making it a crucial target for efforts to improve health [9]. Measuring physical activity is challenging but essential for clinical care and research [10].

Data from self-report instruments such as the International Physical Activity Questionnaire (IPAQ) [11] are associated with BMI [12], but only modestly. Moreover, self-reported physical activity is less accurate than measurements from medical-grade accelerometers such as the ActiGraph [13-16]. Commercial wearable activity monitors or smartwatches such as the Fitbit are growing in popularity and could be used by clinicians and researchers to obtain objective physical activity measurements. They provide step counts that are similar to hand-counted steps [17] and medical-grade devices [16,18,19] and can be used to define minute-to-minute activity intensity [20,21]. Such devices could supplant self-report for physical activity measurement, but they are not designed to capture activities such as biking or swimming that can be self-reported. Several studies have highlighted that compared to activity measurement by self-report, associations between physical activity and BMI are likely stronger when physical activity is measured by objective means by using medical grade accelerometers provided by studies [22-25]. However, no study has explored whether this extends to consumer wearable activity trackers such as Fitbits. This is of particular interest for activities of various intensities where some may not be well-represented on an activity tracker, especially when measuring the same 7 days by activity tracker and self-report.

The Health eHeart Study, which has collected BMI and physical activity information via self-report and Fitbit, provides an opportunity to address this question. We conducted a cross-sectional study to (1) assess the agreement between objective Fitbit-measured physical activity and self-report (IPAQ) and (2) compare the strength of the associations with BMI between Fitbit and IPAQ, utilizing data from the Health eHeart Study [26], a large-scale mobile health study coordinated by the University of California San Francisco. Minute-by-minute Fitbit step counts were used to categorize time by activity intensity for comparisons with analogous self-reported IPAQ measures and calculated measures of overall physical activity from Fitbit (steps/d) and IPAQ (metabolic equivalent of task [MET]/wk). We then compared Fitbit to self-report in terms of cross-sectional associations with BMI. We hypothesized that Fitbit-measured activity would be more strongly associated with BMI than self-reported activity but this would not be consistent across various activity intensities.

## Methods

### Participants in This Study

Health eHeart is a prospective longitudinal cohort study that has enrolled all interested adult volunteers (age≥18 years) who have an email address and have been residing within the United States since March 2013. Participation in the study is entirely web-based and recruitment was conducted on the internet through social media advertisements, email campaigns with advocacy and research organizations, and in person at clinics at the University of California San Francisco. We included participants who completed at least one IPAQ and opted before June 2019 to share data from commercially acquired Fitbits with Health eHeart. Participants were excluded only for missing or invalid data. Health eHeart was approved by the University of California San Francisco Committee on Human Research. Consent was obtained electronically on enrollment. We obtained deidentified data for this study.

### Physical Activity Measurement

Self-reported activity was assessed with the IPAQ short form, which assesses activity at discrete intensities (sedentary, walking, moderate activity, or vigorous activity) over 7 consecutive days [11,12]. Data were cleaned according to the IPAQ protocol with additional criteria developed to address erroneous values (Multimedia Appendix 1). Total active time (sum of time spent walking, moderately active, and vigorously active) and overall activity in weekly MET hours were computed [12].

Physical activity was measured objectively on the same 7 days with Fitbit-brand wearable activity trackers, which are triaxial accelerometers using proprietary algorithms to convert accelerometer counts to minute-by-minute step counts. The Fitbit devices that contributed study data were owned by study participants who obtained their devices outside of the Health eHeart study; the study did not request that participants acquire a Fitbit for this study and did not exclude specific Fitbit device types. Participants received no specific instructions on Fitbit use from Health eHeart. No specific devices were excluded and all were Fitbit brand (Tables S1 and S2 in Multimedia Appendix 1). We defined nonwear time as ≥120 contiguous minutes with 0 steps counted; all other time was considered wear time [27]. We recoded minutes with more than 200 steps as missing, as described previously in accelerometer studies [28,29]. Fitbit data were considered adequate for a given day when there were at least 10 hours of Fitbit wear time during that day. An activity observation was defined as a period of 7 days recalled by a participant on the IPAQ for which the same 7 days had adequate Fitbit data.

We then computed a series of Fitbit-derived physical activity estimates corresponding to standard estimates derived from the IPAQ [30]. Fitbit overall activity was defined as a within-participant average of steps/day during the week. We calculated time spent walking (10-100 steps/min, eg, slow walking), moderately active (101-130 steps/min, eg, brisk walking), vigorously active (131 to 200 steps/min, eg, vigorous walking, jogging, running), and sedentary time, defined as bouts shorter than 120 consecutive minutes with 0-9 steps, to allow for incidental movement [31] and typical sedentary bout lengths [32]. Minutes with at least 10 steps counted were considered active time.

This intensity-defined approach was designed to reflect energy expenditure classifications for the IPAQ [31,33,34]. In a review of literature correlating step rates and METs expended in adults, 64-96 steps/min ranged from 2.0 to 3.1 METs (5 studies), 102-129 steps/min ranged from 2.9 to 5.5 METs (8 studies), and 134 to 170 steps/min ranged from 6.8 to 13.0 METs (7 studies) [35], which are closely aligned with IPAQ definitions of walking (<3 METs), moderate activity (3-6 METs), and vigorous (>6 METs) activity [36,37].

### BMI Measurement

Height, weight, and BMI were self-reported through Health eHeart. Values reported within 90 days of the activity observation were averaged for weight and BMI, and values within 365 days were averaged for height. When both were available, height and weight data were combined to generate a calculated BMI value associated with each activity observation. Calculated BMI values were merged with self-reported BMI values using the median when both were available for an activity observation (supplementary methods in Multimedia Appendix 1).

### Other Measurements

Demographic characteristics, health-related behaviors (ie, alcohol and smoking), and clinical characteristics (ie, history of coronary artery disease, hypertension, hyperlipidemia, and diabetes) were obtained through questionnaires. We used responses within 365 days of and closest to each activity observation.

### Statistical Analyses

Some participants contributed multiple observations over time (Figure S1 in Multimedia Appendix 1). Comparative analyses were restricted to the first activity observation (N=1498). We investigated agreement between IPAQ-measured and Fitbit-measured activity by using Wilcoxon matched-pairs signed-rank test and Spearman’s rank correlations on untransformed data. For the BMI analysis, we excluded participants lacking BMI or covariate data to generate a subsample of 697 observations from 586 participants (Multimedia Appendix 1). We compared normally distributed continuous variables by two-sided *t* test, nonnormal variables by Wilcoxon rank sum test and categorical variables by chi-squared test. Bivariate associations between activity and BMI were examined by Spearman’s rank correlation and nonparametric test-of-trend across heuristically defined physical activity categories at participant level restricted to the first activity observation. We next used multivariable regressions of BMI on Fitbit-measured and IPAQ-measured activity variables by using robust errors clustered by participants to account for activity outliers and nonindependence for participants with multiple observations. Activity distributions and BMI were not transformed. Twelve separate predictor models were fit—one for each of the 6 analogous activity variables measured by either Fitbit or IPAQ. Next, 6 head-to-head combined models were fit with both analogous measurements as predictors (eg, vigorous activity measured by IPAQ and Fitbit in the same model) to assess BMI association strength while controlling for the analogous measurement. All models were adjusted for potential confounding by Fitbit wear-time, device wear location (torso or wrist), and season of data collection and estimated in natural units and in standardized units to account for differences in self-reported and Fitbit-measured activity distributions. We then sequentially adjusted the models for demographic characteristics, health-related behaviors, and clinical characteristics.

We conducted 2 sensitivity analyses to investigate whether data sources affected the main findings of the study. The first examined whether the main findings were sensitive to the inclusion of participants whose data were supplied by MobileTrack (81/1392, 5.8% of comparative analysis cohort; 32/586, 5.5% of BMI analysis subset), which was derived from smartphone measurements and not a wearable activity tracker. The second examined whether the main findings were sensitive to the inclusion of participants whose activity trackers were torso-worn (397/1392, 28.5% of the comparative analysis cohort; 166/586, 28.3% of the BMI analysis subset). For both sensitivity analyses, we excluded the relevant cohort and repeated the study analysis.

Data preparation and analyses were performed using STATA 14 (StataCorp). Two-tailed *P* values less than .05 were considered significant. We report this study according to STROBE (Strengthening the Reporting of Observational Studies in Epidemiology) statement guidelines [38].

## Results

### Comparative Analysis

The age of the participants (N=1498) ranged from 19 to 92 years and they were predominantly Whites (1299/1498, 86.7%), females (895/1498, 59.7%), with at least a bachelor’s degree (1067/1415, 75.4%), and an annual income exceeding US $100,000 (695/1282, 54.2%) (Table 1). All characteristics in Table 1 are summarized at participant level at the first activity observation by using mean (SD) for normal continuous data, median (IQR) for nonnormal continuous, and not available for frequency data.

**Table 1:** Demographic and clinical characteristics of the participants in the comparative analysis and BMI analysis subsets.

Characteristics					Comparative analysis		BMI analysis
**Demographic characteristics**							
	Age (years)^a^, mean (SD)			52.9 (13.9)		51.9 (13.4)	
	Female gender^a^, n (%)			895 (59.8)		370 (63.1)	
	**Education^b^, n (%)**						
		<Bachelor’s degree			348 (24.6)		151 (25.7)
		Bachelor’s degree			492 (34.8)		183 (31.2)
		Postgraduate degree			575 (40.6)		252 (43.0)
	**Annual income (US $)^c^, n (%)**						
		<$50,000			217 (16.9)		99 (16.9)
		$50,000-$100,000			370 (28.9)		175 (29.9)
		>$100,000			695 (54.2)		312 (53.2)
	**Race^d^, n (%)**						
		White			1299 (87.1)		514 (87.7)
		Asian			64 (4.3)		19 (3.2)
		Black/African-American			56 (3.8)		23 (3.9)
		Multiracial			41 (2.8)		17 (2.9)
		Other			32 (2.1)		13 (2.2)
		Hispanic or Latino			82 (5.5)		32 (5.5)
	**Health-related behaviors**						
		**Smoking^e^, n (%)**					
			Never	990 (68.3)		392 (66.9)	
			Past	420 (28.9)		17 (29.5)	
			Current	39 (2.7)		21 (3.6)	
		Alcoholic drinks/wk^f^, median (IQR)			3 (1-7)		3 (1-7)
**Clinical characteristics**							
	Height^g^, (m), median (IQR)			1.70 (1.63-1.78)		1.68 (1.63-1.75)	
	Weight^h^, (kg), median (IQR)			77.6 (66.5-89.4)		77.6 (66.4-8.9)	
	BMI^i^, (kg/m^2^), median (IQR)			26.5 (23.5-30.2)		26.7 (23.5-30.2)	
	Coronary artery disease^j^, n (%)			64 (6.6)		38 (6.5)	
	Diabetes^j^, n (%)			61 (6.3)		36 (6.1)	
	Hyperlipidemia^k^, n (%)			392 (40.7)		240 (41.0)	
	Hypertension^l^, n (%)			347 (35.8)		215 (36.7)	

^a^Calculated for all 1498 comparative analysis participants and 586 BMI analysis participants.

^b^Calculated for 1415 comparative analysis participants and 586 BMI analysis participants.

^c^Calculated for 1282 comparative analysis participants and 586 BMI analysis participants.

^d^Calculated for 1492 comparative analysis participants and 586 BMI analysis participants.

^e^Calculated for 1449 comparative analysis participants and 586 BMI analysis participants.

^f^Available for 1288 comparative analysis participants and 586 BMI analysis participants.

^g^Available for 1076 comparative analysis participants and 533 BMI analysis participants.

^h^Available for 1095 comparative analysis participants and 546 BMI analysis participants.

^i^Available for 1196 comparative analysis participants and 586 BMI analysis participants.

^j^Available for 968 comparative analysis participants and 586 BMI analysis participants.

^k^Available for 963 comparative analysis participants and 586 BMI analysis participants.

^l^Available for 967 comparative analysis participants and 586 BMI analysis participants.

Physical activity observations were not evenly distributed among seasons (Wald test, *P*<.001) and 995 (71.5%) of 1392 Fitbit devices were wrist-worn (Table 2). In Table 2, all characteristics are summarized at participant level at the first activity observation by using mean (SD) for normal continuous data, median (IQR) for nonnormal continuous, and not available for frequency data. There were no missing data for the BMI analysis. The median overall activity at the first activity observation was 35.5 MET-h/wk (IQR 18.1-62.2) by IPAQ. Fitbits counted a median of 8622 steps/d (IQR 6191-11061) over the same week. Compared to Fitbit measurements, participants self-reported more moderate activity (median of 90 min/wk vs 75 min/wk, Wilcoxon matched-pairs sign-rank test *P*<.001) and vigorous activity (80 min/wk vs 0 min/wk, *P*<.001), but self-reported less time spent sedentary (42.0 h/wk vs 87.2 h/wk; *P*<.001) and walking (3.5 h/wk vs 20.2 h/wk, *P*<.001).

**Table 2:** Physical activity characteristics of the participants in the comparative analysis and BMI analysis subset.

Characteristics		Comparative analysis	BMI analysis subset
**Season of data collection^a^, n (%)**			
	Spring	340 (22.7)	161 (27.5)
	Summer	445 (29.7)	149 (25.4)
	Fall	323 (21.6)	141 (24.1)
	Winter	390 (26.0)	135 (23.0)
**Fitbit**			
	Wearing on wrist^b^, n (%)	995 (71.5)	420 (71.7)
	Wearing on torso^b^, n (%)	397 (28.5)	166 (28.3)
	Wear time (h/d)^a^, median (IQR)	17.1 (16.0-18.5)	17.1 (15.9-18.5)
	Overall activity (steps/d)^a^, median (IQR)	8622 (6191-11,061)	8778 (6385-11,115)
	Active time (h/wk)^a^, median (IQR)	22.9 (18.0-28.0)	23.1 (18.5-28.1)
	Sedentary (h/wk)^a^, median (IQR)	87.2 (80.5-94.2)	87.3 (80.7-94.9)
	Walking (h/wk)^a^, median (IQR)	20.2 (15.9-25.0)	20.8 (16.6-25.5)
	Moderate activity (min/wk)^a^, median (IQR)	75 (26-163)	75 (28-166)
	Vigorous activity (min/wk)^a^, median (IQR)	0 (0-11)	1 (0-12)
**IPAQ^a,c^**			
	Overall activity (metabolic equivalent of tasks-h/wk), median (IQR)	35.5 (18.1-62.2)	34.9 (19.3-60.0)
	Active time (h/wk), median (IQR)	8.0 (4.3-14.0)	8.2 (4.5-13.2)
	Sedentary (h/wk), median (IQR)	42.0 (21.0-56.0)	42.0 (26.8-56.0)
	Walking (h/wk), median (IQR)	3.5 (2.0-7.0)	3.5 (1.7-7.0)
	Moderate activity (min/wk), median (IQR)	90 (20-200)	90 (30-200)
	Vigorous activity (min/wk), median (IQR)	80 (0-180)	80 (0-180)

^a^Calculated for 1498 comparative analysis participants and 586 BMI analysis participants.

^b^Calculated for 1345 comparative analysis participants and 586 BMI analysis participants.

^c^IPAQ: International Physical Activity Questionnaire.

**Figure 1:**
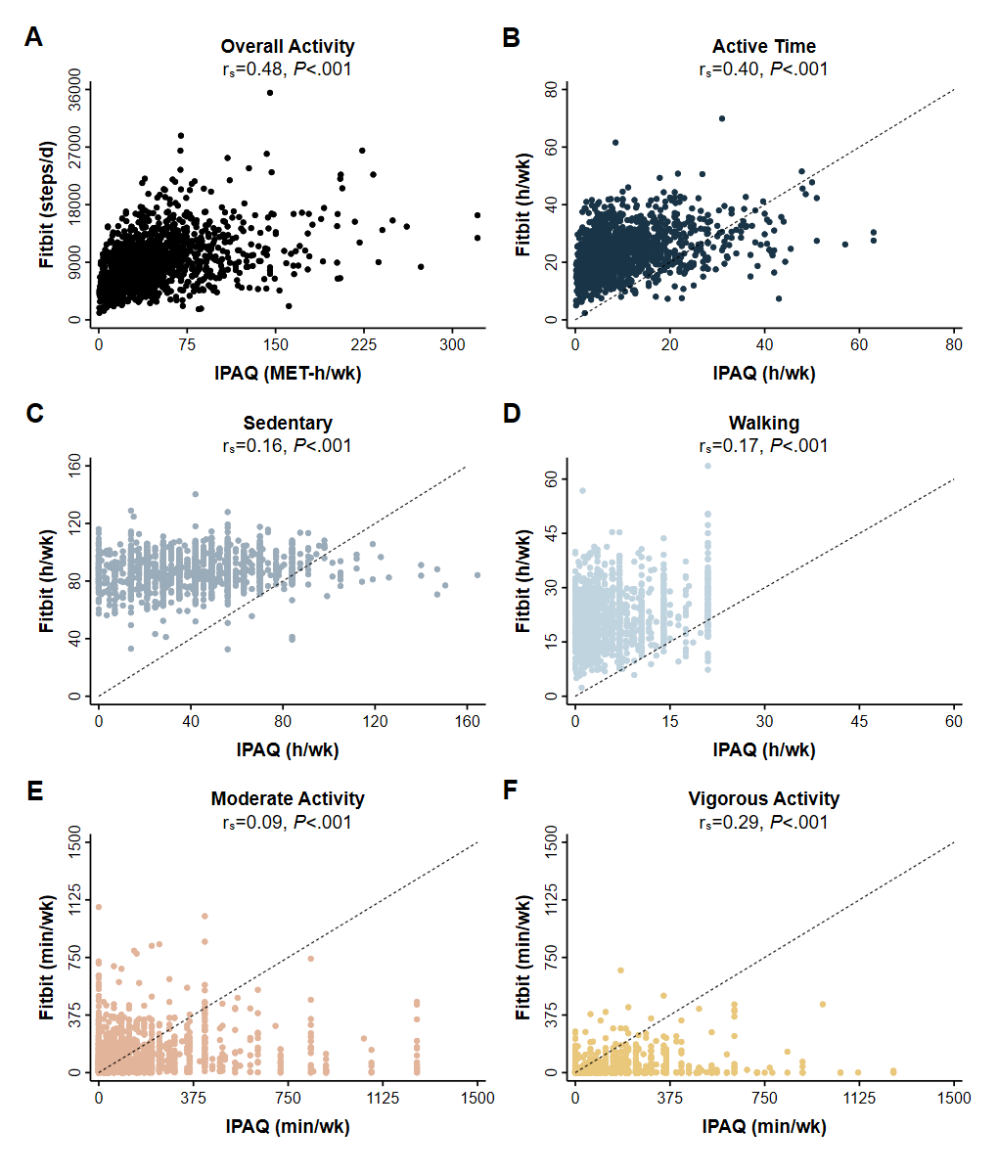
Fitbit-measured and self-reported physical activity of 1498 participants. Each symbol represents a participant’s first paired activity observation. Spearman’s rank correlations (rs) and *P* values are provided for the following: A. Overall activity and intensity-defined time spent. B. Active in total (sum of walking, moderate activity, and vigorous activity). C. Sedentary D. Walking E. Moderately active, and F. Vigorously active. Fitbit measurements were acquired during the same period queried by self-report (IPAQ). Dashed lines represent identical measurements. Overall activity was measured on different scales (Fitbit, steps/d; IPAQ, MET-h/wk) and no dashed line is provided. IPAQ: International Physical Activity Questionnaire; MET: metabolic equivalent of task.

Overall activity measurements by Fitbit (steps/d) and IPAQ (MET-h/wk) were moderately correlated (Spearman’s rank r_s_=0.48), as was total active time (r_s_=0.40) and vigorous activity (r_s_=0.29) (Figure 1, Figure S2 in Multimedia Appendix 1). By contrast, time spent sedentary, walking, or moderately active were weakly correlated (walking r_s_=0.17, sedentary r_s_=0.16, moderate activity r_s_=0.09). Findings were very similar when we excluded participants whose data source was the MobileTrack app, and when we excluded participants whose data were obtained from an activity tracker worn on the torso.

### BMI Analysis

The subset of participants with a BMI measurement was included in the BMI analysis sample (n=586). In bivariate analyses using categorical physical activity, Fitbit measurements of activity were significantly associated with BMI for all intensity-defined (Table 3) and overall activity measurements (Table 4) (*P* values range from <.001 to .01), whereas overall activity, total active time, sedentary time, and walking time measured by IPAQ were significantly associated with BMI (*P* values range from .001 to .01).

**Table 3:** Activity distributions and bivariate associations with BMI by activity intensity in the BMI analysis subset (n=586)^a^.

Fitbit								IPAQ^b^								
Time (h/wk)			n (%)		BMI, mean (SD)		*P* value		Time (h/wk)		n (%)		BMI, mean (SD)			*P* value
**Sedentary**						.01								.001		
	0-19	0		N/A^c^				0-19		101 (17.2)		26.4 (5.2)				
	20-39	0		N/A				20-39		159 (27.1)		26.9 (5.0)				
	40-59	4 (0.7)		28.2 (7.8)				40-59		197 (33.6)		28.0 (6.2)				
	60-79	133 (22.7)		26.5 (5.7)				60-79		93 (15.9)		28.1 (6.0)				
	80+	449 (76.6)		28.0 (6.0)				80+		36 (6.1)		31.0 (8.6)				
**Walking**						.001								.01		
	0-4	0		N/A				0-4		357 (60.9)		28.0 (6.0)				
	5-9	19 (3.2)		30.4 (7.8)				5-9		136 (23.2)		27.3 (5.5)				
	10-14	79 (13.5)		28.8 (6.8)				10-14		49 (8.4)		27.6 (7.6)				
	15-19	170 (29.0)		28.4 (6.6)				15-19		12 (2.0)		26.6 (5.3)				
	20-24	158 (27.0)		27.3 (6.0)				20-24		32 (5.5)		25.4 (4.8)				
	25+	160 (27.3)		26.3 (4.0)				25+		0		N/A				
**Moderate activity**						<.001								.19		
	0-1	241 (41.1)		28.7 (6.4)				0-1		194 (33.1)		28.3 (7.0)				
	1-2	134 (22.9)		27.5 (5.1)				1-2		113 (19.3)		27.3 (5.9)				
	2-3	82 (14.0)		26.8 (5.4)				2-3		90 (15.4)		27.8 (5.2)				
	3-4	53 (9.0)		26.8 (5.5)				3-4		64 (10.9)		27.3 (5.2)				
	4-5	26 (4.4)		25.5 (4.4)				4-5		33 (5.6)		27.3 (4.9)				
	5+	50 (8.5)		26.0 (7.1)				5+		92 (15.7)		26.8 (5.0)				
**Vigorous activity**						<.001								.13		
	0-1	534 (91.1)		27.9 (5.9)				0-1		249 (42.5)		28.4 (6.5)				
	1-2	29 (4.9)		26.5 (8.2)				1-2		99 (16.9)		27.4 (6.5)				
	2-3	13 (2.2)		22.8 (1.9)				2-3		84 (14.3)		26.9 (5.7)				
	3-4	5 (0.9)		23.7 (2.5)				3-4		43 (7.3)		26.4 (3.6)				
	4-5	3 (0.5)		25.8 (7.7)				4-5		44 (7.5)		28.2 (5.6)				
	5+	2 (0.3)		24.0 (1.9)				5+		67 (11.4)		26.6 (4.2)				

^a^Data were restricted to the first activity observation (586 observations). *P* values are for nonparametric test-of-trend across heuristically defined categories of time spent at each level of activity.

^b^IPAQ: International Physical Activity Questionnaire.

^c^N/A: not applicable.

**Table 4:** Overall activity distributions and bivariate associations with BMI in the BMI analysis subset (n=586)^a^.

Fitbit								IPAQ^b^								
Time (h/wk)			n (%)		BMI, mean (SD)		*P* value		Time (h/wk)		n (%)		BMI, mean (SD)			*P* value
**Total active time**						<.001								.008		
	0-9	8 (1.4)		32.7 (8.2)				0-9		366 (62.5)		28.2 (6.2)				
	10-19	184 (31.4)		29.1 (7.1)				10-19		153 (26.1)		26.8 (5.5)				
	20-29	285 (48.6)		27.2 (5.0)				20-29		46 (7.8)		26.9 (6.1)				
	30-39	99 (16.9)		26.1 (5.7)				30-39		12 (2.0)		26.8 (5.3)				
	40+	10 (1.7)		25.3 (3.1)				40+		9 (1.5)		25.1 (3.0)				
**Overall activity, (steps/d)**						<.001								^c^		
	0-2999	14 (2.4)		31.0 (8.1)				^c^		^c^		^c^				
	3000-5999	107 (18.3)		30.6 (7.8)				^c^		^c^		^c^				
	6000-8999	191 (32.6)		27.6 (5.0)				^c^		^c^		^c^				
	9000-11,999	166 (28.3)		26.9 (4.7)				^c^		^c^		^c^				
	12,000-14,999	70 (11.9)		25.5 (4.8)				^c^		^c^		^c^				
	15,000+	38 (6.5)		25.4 (7.2)				^c^		^c^		^c^				
**Overall activity, (MET-h/wk)**						^c^								.006		
	^c^	^c^		^c^				0-19		155 (26.4)		29.2 (7.4)				
	^c^	^c^		^c^				20-39		181 (30.9)		27.3 (5.1)				
	^c^	^c^		^c^				40-59		103 (17.6)		27.2 (5.0)				
	^c^	^c^		^c^				60-79		68 (11.6)		26.5 (6.3)				
	^c^	^c^		^c^				80-99		32 (5.5)		26.0 (4.1)				
	^c^	^c^		^c^				100+		47 (8.0)		27.3 (5.5)				

^a^Data are restricted to the first activity observation (586 observations). *P* values are for nonparametric test-of-trend across heuristically defined categories of time spent at each level of activity.

^b^IPAQ: International Physical Activity Questionnaire.

^c^Cells omitted because overall activity was measured on different scales (Fitbit, steps/d; IPAQ, MET-h/wk).

**Figure 2:**
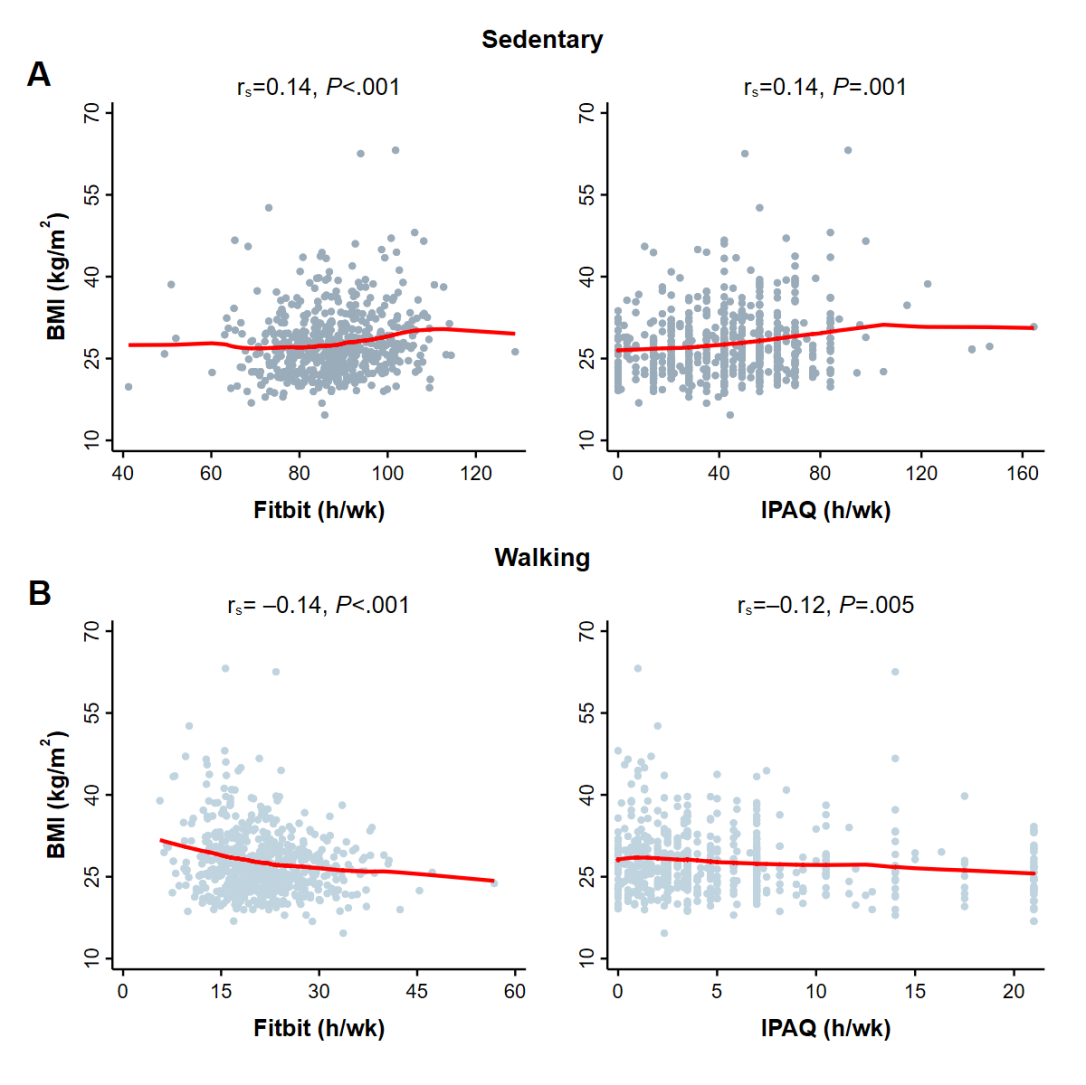
BMI correlations for Fitbit and IPAQ-measured sedentary and walking time of 586 participants. Each symbol represents a participant’s first paired activity observation. Spearman’s rank correlations (rs) and *P* values are provided for time spent in A. Sedentary and B. Walking. Fitbit measurements were acquired during the same period queried by self-report (IPAQ). Solid red lines are results of locally weighted regressions. Overall activity was measured on different scales (Fitbit, steps/d; IPAQ, MET-h/wk). IPAQ: International Physical Activity Questionnaire; MET: metabolic equivalent of task.

**Figure 3:**
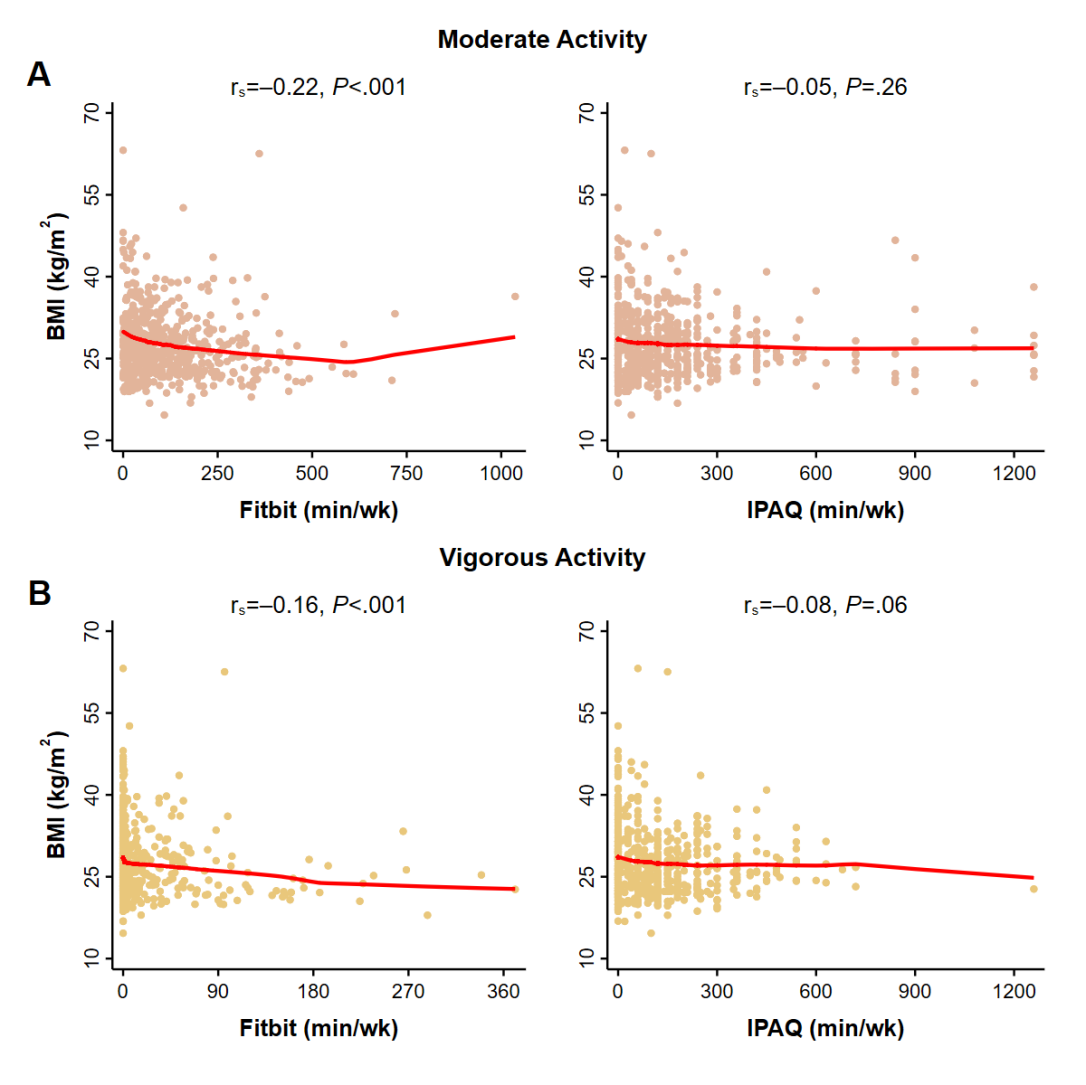
BMI correlations for Fitbit and IPAQ-measured moderate and vigorous activity of 586 participants. Each symbol represents a participant’s first paired activity observation. Spearman’s rank correlations (rs) and *P* values are provided for A. Moderate activity and B. Vigorous activity. Fitbit measurements were acquired during the same period queried by self-report (IPAQ). Solid red lines are results of locally weighted regressions. Overall activity was measured on different scales (Fitbit, steps/d; IPAQ, MET-h/wk). IPAQ: International Physical Activity Questionnaire; MET: metabolic equivalent of task.

**Figure 4:**
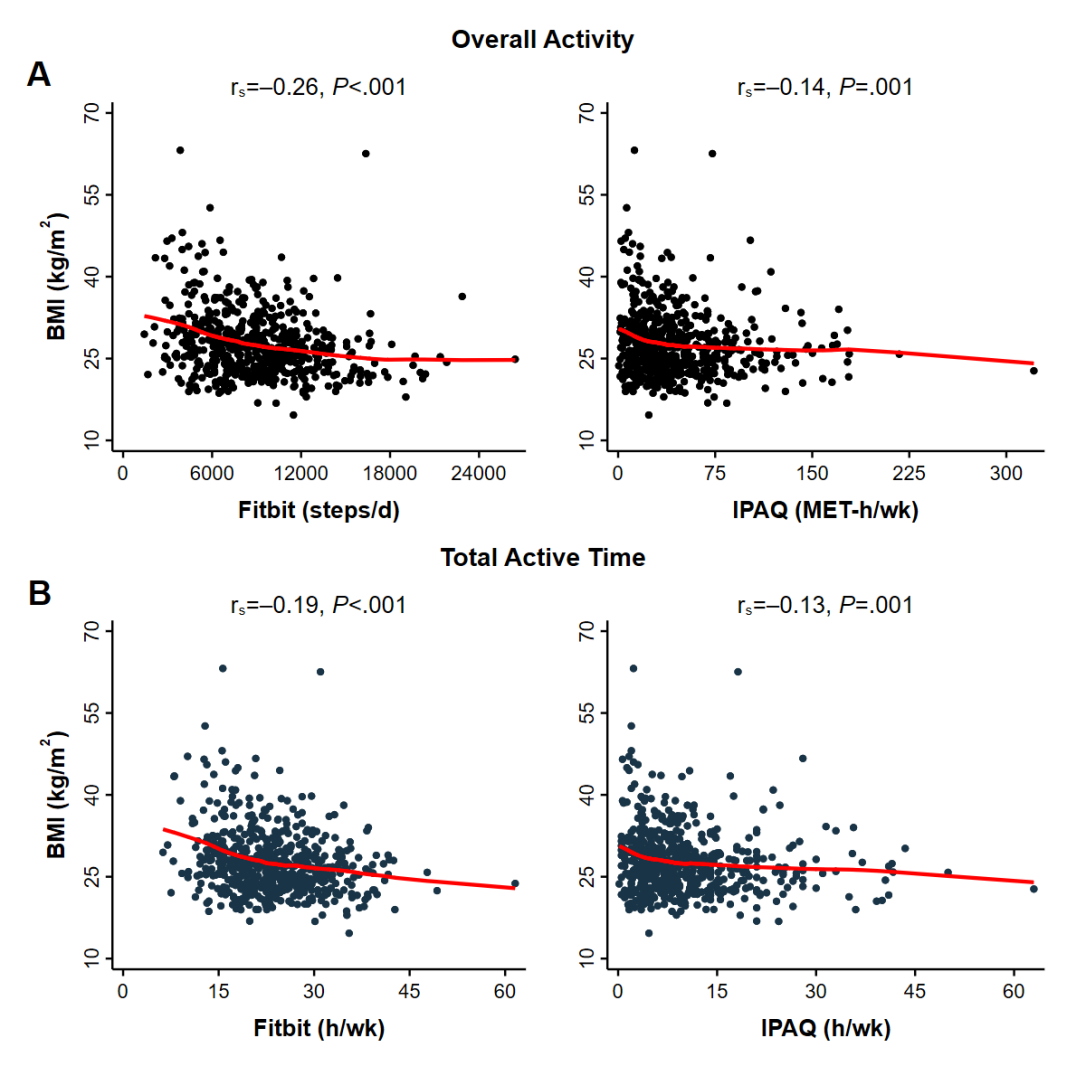
BMI correlations for Fitbit and IPAQ-measured overall activity and total active time of 586 participants. Each symbol represents a participant’s first paired activity observation. Spearman’s rank correlations (rs) and *P* values are provided for A. Overall activity and B. Active in total (sum of walking, moderate activity, and vigorous activity). Fitbit measurements were acquired during the same period queried by self-report (IPAQ). Solid red lines are results of locally weighted regressions. Overall activity was measured on different scales (Fitbit, steps/d; IPAQ, MET-h/wk). IPAQ: International Physical Activity Questionnaire; MET: metabolic equivalent of task.

All Fitbit measurements, including those of lower intensity activities (Figure 2), higher intensity activities (Figure 3), and overall activity (Figure 4) were correlated with BMI (Spearman’s rank test, 6 comparisons, all *P*<.001). By contrast, IPAQ measurements of lower intensity activity (Figure 2) and overall activity (Figure 4) were correlated with BMI (*P* values range from .001 to .005) but not the higher intensity moderate activity (*P*=.26) or vigorous activity (*P*=.06) (Figure 3).

After adjustment for demographics, health-related behaviors, and clinical characteristics, an increase of 1 h/wk in Fitbit-measured vigorous activity was associated with a BMI that was 0.84 kg/m^2^ lower (95% CI –1.35 to –0.32, *P*<.001) (Table 5). By contrast, an increase of 1 h/wk in self-reported vigorous activity on the IPAQ was associated with a BMI that was 0.17 kg/m^2^ lower (95% CI –0.34 to –0.00, *P*=.045), which was significantly smaller than the coefficient for Fitbit-measured vigorous activity (*P*=.01).

**Table 5:** BMI associations for Fitbit versus International Physical Activity Questionnaire activity by intensity in multivariable logistic regressions^a^.

Activity predictor			Unadjusted		Adjusted for demographics		Adjusted for demographics, health-related behaviors, and clinical characteristics	
			Coefficient (95% CI)	*P* value	Coefficient (95% CI)	*P* value	Coefficient (95% CI)	*P* value
**Sedentary** **(h/wk)**								
	**Separate predictor models**			.11		.16		.16
		Fitbit	0.09^b^ (0.02 to 0.15)		0.09^b^ (0.03 to 0.15)		0.08^b^ (0.02 to 0.14)	
		IPAQ^c^	0.04^d^ (0.02 to 0.06)		0.05^d^ (0.03 to 0.07)		0.04^d^ (0.02 to 0.06)	
	**Combined predictor model**			.35		.61		.58
		Fitbit	0.07^e^ (0.00 to 0.13)		0.06 (–0.00 to 0.12)		0.06 (–0.00 to 0.12)	
		IPAQ	0.03^b^ (0.01 to 0.06)		0.04^d^ (0.02 to 0.06)		0.04^d^ (0.02 to 0.06)	
**Walking** **(h/wk)**								
	**Separate predictor models**			.30		.35		.30
		Fitbit	–0.20^d^ (–0.27 to –0.13)		–0.19^d^ (–0.27 to –0.12)		–0.18^c^ (–0.24 to –0.11)	
		IPAQ	–0.15^b^ (–0.23 to –0.06)		–0.15^d^ (–0.23 to –0.07)		–0.12^b^ (–0.20 to –0.04)	
	**Combined predictor model**			.20		.24		.22
		Fitbit	–0.18^d^ (–0.26 to –0.11)		–0.18^d^ (–0.25 to –0.10)		–0.16^d^ (–0.23 to –0.09)	
		IPAQ	–0.10^e^ (–0.19 to –0.01)		–0.10^e^ (–0.19 to –0.02)		–0.08^e^ (–0.17 to 0.00)	
**Moderate activity** **(h/wk)**								
	**Separate predictor models**			.01		.04		.06
		Fitbit	–0.50^d^ (–0.79 to –0.22)		–0.44^d^ (–0.71 to –0.17)		–0.41^b^ (–0.68 to –0.15)	
		IPAQ	–0.11 (–0.23 to 0.01)		–0.13^e^ (–0.25 to –0.01)		–0.13^e^ (–0.25 to –0.02)	
	**Combined predictor model**			.02		.06		.08
		Fitbit	–0.49^b^ (–0.77 to –0.21)		–0.42^b^ (–0.70 to –0.15)		–0.40^b^ (–0.66 to –0.13)	
		IPAQ	–0.08 (–0.20 to 0.03)		–0.11 (–0.23 to 0.01)		–0.11 (–0.23 to 0.00)	
**Vigorous activity** **(h/wk)**								
	**Separate predictor models**			<.001		<.001		.01
		Fitbit	–1.19^d^ (–1.70 to –0.68)		–1.04^d^ (–1.52 to –0.56)		–0.84^b^ (–1.35 to –0.32)	
		IPAQ	–0.21^e^ (–0.38 to –0.05)		–0.21^e^ (–0.37 to –0.04)		–0.17^e^ (–0.34 to –0.00)	
	**Combined predictor model**			<.001		.003		.03
		Fitbit	–1.10^c^ (–1.61 to –0.59)		–0.95^d^ (–1.43 to –0.47)		–0.76^b^ (–1.28 to –0.25)	
		IPAQ	–0.16 (–0.32 to 0.00)		–0.16 (–0.32 to 0.00)		–0.14 (–0.30 to 0.03)	

^a^All models were adjusted for Fitbit wear-time, data collection season, and Fitbit device wear location. Demographics include age, gender, education, income, race, and ethnicity. Health-related behaviors include smoking and alcohol use. Clinical characteristics include coronary artery disease, diabetes, hyperlipidemia, and hypertension. This table shows the BMI difference (kg/m^2^) per predictor increase (95% CI). Regressions accounted for multiple observations with robust standard errors clustered by participant. *P* values shown are for Wald tests comparing Fitbit versus IPAQ coefficients.

^b^These values were significant at *P*<.01 for cluster robust association with BMI (586 participant clusters).

^c^IPAQ: International Physical Activity Questionnaire.

^d^These values were significant at *P*<.001 for cluster robust association with BMI (586 participant clusters).

^e^These values were significant at *P*<.05 for cluster robust association with BMI (586 participant clusters).

In fully adjusted combined models with both analogous Fitbit and IPAQ measurements in the same model (eg, vigorous activity measured by Fitbit and IPAQ included as predictors in the same model), coefficients displayed a similar pattern of magnitude to the fully-adjusted single predictor models (Table 5). However, only Fitbit measurements were significantly associated with BMI in combined head-to-head models for moderate and vigorous activity, whereas the reverse was the case for sedentary time.

When using standardized units to account for differences in the typical ranges between self-reported and Fitbit-measured activity using fully-adjusted single predictor models, magnitudes of association with BMI were larger for Fitbit versus IPAQ-measured time spent walking, moderately active, or vigorously active, but these did not reach statistical significance (Table S5 in Multimedia Appendix 1).

In separate regressions, an increase in Fitbit-measured overall activity of 1 SD (3663 steps/d) was associated with a BMI that was 1.37 kg/m^2^ lower after adjusting for demographic factors, health-related behaviors, and clinical characteristics (β=–1.37, 95% CI –1.84 to –0.90; *P*<.001), whereas 1 SD increase in IPAQ-measured overall activity (37.2 MET-h/wk) was associated with a BMI that was 0.72 kg/m^2^ lower (β=–.72, 95% CI –1.14 to –0.29; *P*=.002) (Table 6, complete regression results in Table S3 in Multimedia Appendix 1). In Table 6, overall activity units were standardized due to their different scales (natural unit analyses of overall activity are presented in Table S4 in Multimedia Appendix 1). Findings were very similar when we excluded participants whose data source was the MobileTrack app and when we excluded participants whose data were obtained from an activity tracker worn on the torso.

**Table 6:** BMI associations for Fitbit versus IPAQ by overall activity and total active time in multivariable logistic regressions^a^.

Activity predictor				Unadjusted				Adjusted for demographics				Adjusted for demographics, health-related behaviors, and clinical characteristics		
				Coefficient (95% CI)		*P* value		Coefficient (95% CI)		*P* value		Coefficient (95% CI)		*P* value
**Overall activity per 1 SD/wk^b^**														
	**Separate predictor models**				.001				.007				.01	
		Fitbit	–1.65^c^ (–2.15 to –1.16)				–1.51^c^ (–1.99 to –1.03)				–1.37^c^ (–1.84 to –0.90)			
		IPAQ^d^	–0.80^e^ (–1.23 to –0.35)				–0.82^c^ (–1.24 to –0.39)				–0.72^e^ (–1.14 to –0.29)			
	**Combined predictor model**				.001				.005				.01	
		Fitbit	–1.57^c^ (–2.09 to –1.05)				–1.40^c^ (–1.90 to –0.89)				–1.28^c^ (–1.78 to –0.77)			
		IPAQ	–0.24 (–0.66 to 0.19)				–0.30 (–0.72 to 0.12)				–0.25 (–0.69 to 0.19)			
**Total active time (h/wk)**														
	**Separate predictor models**				<.001				<.001				.002	
		Fitbit	–0.23^c^ (–0.30 to –0.16)				–0.22^c^ (–0.28 to –0.15)				–0.20^c^ (–0.26 to –0.13)			
		IPAQ	–0.10^c^ (–0.15 to –0.04)				–0.10^c^ (–0.15 to –0.05)				–0.09^e^ (–0.14 to –0.04)			
	**Combined predictor model**				.001				.002				.006	
		Fitbit	–0.21^c^ (–0.28 to –0.14)				–0.20^c^ (–0.27 to –0.13)				–0.18^c^ (–0.25 to –0.11)			
		IPAQ	–0.04 (–0.10 to 0.01)				–0.05 (–0.10 to 0.01)				–0.04 (–0.09 to 0.01)			

^a^All models were adjusted for Fitbit wear-time, data collection season, and Fitbit device wear location. Demographics include age, gender, education, income, race, and ethnicity. This table shows the BMI difference (kg/m^2^) per predictor increase (95% CI). Health-related behaviors include smoking and alcohol use. Clinical characteristics include coronary artery disease, diabetes, hyperlipidemia, and hypertension. Regressions accounted for multiple observations with robust standard errors clustered by participant. *P* values shown are for Wald tests comparing Fitbit versus IPAQ coefficients.

^b^1 SD for overall activity was 3663 steps/d for Fitbit and 37.2 MET-h/wk for International Physical Activity Questionnaire.

^c^These values were significant at *P*<.001 for cluster robust association with BMI (586 participant clusters).

^d^IPAQ: International Physical Activity Questionnaire.

^e^These values were significant at *P*<.01 for cluster robust association with BMI (586 participant clusters).

## Discussion

In this study of adults who owned a Fitbit and enrolled in the web-based Health eHeart study, we found that physical activity measured by a wearable activity tracker (Fitbit) was not strongly correlated with self-reported activity but generally proved to be more strongly associated than self-reported activity with a key marker of cardiometabolic health—BMI. Fitbit and self-reported activity measurements were moderately correlated for overall activity, total active time, and nearly so for vigorous activity, but only weakly correlated for sedentary time, walking, and moderate activity. Participants self-reported more vigorous activity, slightly more moderate activity, and less sedentary and walking time than was measured by Fitbit. Associations with BMI were typically stronger for Fitbit compared to self-reported activity from the IPAQ, particularly for higher intensity activities (moderate and vigorous activity) and summary measures (time spent actively and steps/d), both in separate regression models and head-to-head combined models.

Our study found only modest correlations between self-report and device data, a phenomenon that has been previously described [39,40]. The stronger moderate correlation observed for overall activity between Fitbit and IPAQ (r_s_=0.48) agrees with previously reported values of correlations between self-report and device data for general physical activity measurements [13].

Our models estimated that Fitbit-measured steps/d, a common measure of overall physical activity derived from wearable activity trackers, was associated with about twice as much difference in BMI as self-reported MET-h/wk, the IPAQ summary measure encapsulating overall physical activity. This is consistent with 3 recent studies using medical-grade accelerometers that found stronger associations with BMI compared to self-report—one in a representative sample of over 4700 adults in the United States [22], one of nearly 500 adults in Malaysia [23], and a third of 317 in Chile that also demonstrated stronger associations with other cardiometabolic risk markers such as fasting glucose and lipid levels [25]. Our twofold larger association also approximates that found in a study of over 80,000 participants in the United Kingdom, in which there was typically a 5-6-year discrepancy between self-report and accelerometer data collection [24]. Our study is the first such analysis to estimate comparative associations with BMI by using activity measurements acquired from consumer wearable activity trackers under free-living conditions that were collected during the same period of time, providing a within-subject control for activities performed. The larger coefficient for the activity tracker measures in this study thus cannot by explained by differences in the actual activity levels between data collection for the wearable devices and self-report. The effect magnitudes estimated in this study are also consistent with studies estimating the activity-BMI relationship separately using objective and self-report methods [12,41].

Across all standardized unit activity measurements from both Fitbit and self-report, Fitbit steps/d was associated with a substantial difference in BMI per SD increase (β=–1.37, *P*<.001). Measurements from Fitbit but not IPAQ remained significantly associated with BMI in regression models that were specified with both for overall activity, total active time, moderate activity, and vigorous activity, thereby suggesting that Fitbit data captures BMI-relevant variability in physical activity better than self-report. Taken together, these findings suggest that Fitbits are advantageous in comparison to self-report primarily because they provide more informative measurements of moderate-to-vigorous physical activity and overall physical activity (steps/d or total active time).

If we assume that these Fitbit associations reflect entirely causal effects of physical activity on BMI (ie, no residual confounding), the impact of Fitbit-measured physical activity can be described in terms that could be useful for clinician-patient interactions and other purposes. For example, 1 SD increase in steps/d measured by Fitbit (3663 steps/d) was associated with a BMI that was 1.37 kg/m^2^ lower in this study. Such step increases are reasonably attainable given that prospective interventions with activity trackers have demonstrated typical step/d increases of about 2500 [42,43]. It is worth noting, though, that more modest decreases in BMI are typically observed in prospective observational studies and trials using activity tracker interventions [43,44], and the relationship between BMI and physical activity when using a wearable activity tracker is still unclear. For example, a recent randomized trial providing obese participants with a diet and exercise intervention along with a wearable activity tracker found that the group receiving the device lost significantly less weight over 2 years [45]. However, the techniques to increase physical activity most effectively and otherwise modify behavior to induce healthy weight loss remains unclear [46].

This study has several limitations. Although our participants self-reported physical activity levels that were similar to those mentioned in previous multinational studies (about 36 MET-h/wk) [11], our Fitbit-measured levels of activity (median 8622 steps/d) were higher than previous estimates from nationally representative samples (about 6500 steps/d) [29]. This likely reflects healthy volunteer bias in the Health eHeart Study, but associations with BMI should still be valid given the range of activity levels reflected in the study. The high step counts in this study might also reflect a bias toward increased device adherence when exercising more often than usual, although such systematic overestimation might not affect associations with BMI. Generalizability is limited by the sample’s demographic homogeneity and intrinsic self-selection biases. For example, individuals who self-select to use an activity tracker consistently over time such as our participants likely differ in unclear ways from those who abandon the device rapidly or use it infrequently. Participants could receive feedback on activity via their Fitbit, which could influence recall for the IPAQ and bias estimate comparisons. We conducted a large number of analyses and presented *P* values for many of them to illustrate the potential role of chance; it is important for readers to note that we did not correct for multiple comparisons, on the grounds that our hypotheses are separable and not merely equivalent paths to achieving statistical equivalence for a single global hypothesis test. However, it is certainly possible that one or more of the statistically significant findings that we observed occurred by chance. Lastly, we recognize that step-counting algorithms have evolved over the study period, but the predictive advantages of wearables will likely increase over time as algorithms improve and become more accurate.

Objective measurements of moderate and vigorous activity that were less than self-reported values could reflect a wearable activity tracker’s inability to detect activities such as biking or user behavior such as removing a device before swimming, but failing to capture these activities could be expected to weaken BMI associations for Fitbit-derived measures only and would not account for the larger BMI associations for Fitbit in this study. Future studies comparing objective and subjective measurements of activity using commercially available accelerometers designed to also capture such activities would help clarify this point.

In summary, our study provides evidence that wearable activity trackers such as Fitbits provide information about physical activity that is likely more clinically meaningful than self-report. The health benefits of physical activity span numerous disease processes responsible for significant morbidity and mortality [47] such that improving physical activity measurement could significantly impact public health. As the risk attributable to physical inactivity and obesity from noncommunicable diseases such as coronary heart disease continues to grow worldwide [48,49], objective measurements of physical activity from Fitbit and similar wearable activity trackers may play an increasingly important role in improving health through physical activity measurement [50].

In conclusion, in this cross-sectional analysis of Health eHeart Study participants using their Fitbit devices in free-living conditions, step measurements from Fitbits were more strongly associated with BMI than self-reported activity from the same period, particularly for higher intensity activity and summary measures of activity. Fitbit measurements were only moderately or weakly correlated with self-reported physical activity. Wearable activity trackers such as Fitbit likely provide more meaningful data than self-report with respect to weight for clinicians, researchers, and patients.

## Appendix

Multimedia Appendix 1 Supplementary materials.

## Abbreviations

IPAQ: International Physical Activity Questionnaire
MET: metabolic equivalent of task
