# Supplementary material for: Comparison of the Physical Activity Measured by a Consumer Wearable Activity Tracker and That Measured by Self-Report: Cross-Sectional Analysis of the Health eHeart Study
Source: JMIR Mhealth Uhealth. 2020 Dec 29;8(12):e22090. doi: 10.2196/22090 (PMC7803477; doi:10.2196/22090)
Supplement: Multimedia Appendix 1 [file mhealth_v8i12e22090_app1.docx]

**Supplementary Materials Contents**

[Supplementary Methods 2](#_Toc57661425)

[Measurements and data cleaning 2](#_Toc57661426)

[Supplementary Results 4](#_Toc57661427)

[Comparative analysis 4](#_Toc57661428)

[Supplementary References 5](#_Toc57661429)

[Supplementary Figures 6](#_Toc57661430)

[Figure S1. Participant selection diagram for the two study samples. 6](#_Toc57661431)

[Figure S2. Spearman rank correlations between all physical activity measurements acquired from Fitbits and self-report (IPAQ) for the 1,498 participants in the comparative analysis. 7](#_Toc57661432)

[Supplementary Tables 8](#_Toc57661433)

[Table S1. 8](#_Toc57661434)

[Table S2. 9](#_Toc57661435)

[Table S3. 10](#_Toc57661436)

[Table S4. 13](#_Toc57661437)

[Table S5. 14](#_Toc57661438)

# **Supplementary Methods**

## Measurements and data cleaning

The International Physical Activity Questionnaire (IPAQ) queried activities performed at various intensities, including time spent walking, moderately active (e.g., carrying light loads, bicycling at a regular pace, or doubles tennis), and vigorously active (e.g., heavy lifting, digging, aerobics, or fast bicycling). Typical bout lengths for vigorous activity, moderate activity, and walking time were multiplied by the number of days those activities occurred during the week queried to generate a variable indicating the total time spent at each activity intensity. We then defined total activity as the total time per week spent vigorously active, moderately active, and walking. Participants also indicated time spent sedentary on a typical day, from which total sedentary time was computed by multiplying the typical value by seven. In keeping with IPAQ data cleaning guidelines,^1^ we truncated the number of minutes spent walking, moderately active, or vigorously active at 180 minutes for each intensity. IPAQ surveys were excluded if they met criteria defined in the protocol, namely if total activity exceeded the equivalent of 16 hrs/day (n=0). We developed additional criteria to remove values that appeared erroneous among the 4,028 remaining questionnaires. These included questionnaires with no time reported in any activity category (n=548), active time of zero minutes (n=119), and observations where the total of time across all categories exceeded the equivalent of 24 hrs/day (n=22). A summary measure of overall physical in units of MET-hrs/week was computed according to IPAQ scoring protocols using the formula 3.3 × walking hrs/week + 4 × moderate activity hrs/week + 8 × vigorous activity hrs/week.

Fitbit data was stored on a cloud-based server and shared to Health eHeart when participants consented to do so by authorizing an authenticated (https://oauth.net/2/) server-to-server communication between the Health eHeart Study and Fitbit. Once the connection was established, all data collected by all Fitbit brand devices since purchase and the first use of the device were available for study analyses.

Income and education data were measured as categorical variables and recoded into three levels for clarity (income: less than $50,000, $50,000 to $100,00, greater than $100,000 annually; educational attainment: less than a bachelor’s degree, bachelor’s degree, postgraduate degree). Smoking history was recoded to a three-level categorical variable indicating whether participants had never smoked, smoked regularly in the past, or were currently smoking. Alcohol use was measured as a continuous variable indicating the number of standard drinks the participant consumed in a typical week.

BMI data was measured by self-report and also generated as a composite variable from raw height and weight data. Raw weights less than 20 kg were considered physiologically unreasonable and recoded as missing. Heights that were reported within 365 days of each physical activity observation were then averaged to generate a composite height for that activity observation. Weights within 90 days of the observation were similarly averaged. These values were then leveraged to compute a calculated BMI associated with each activity observation using the formula kg/m^2^. Self-reported BMI values within 90 days of each activity observation were then averaged to generate a self-report average BMI time-linked to each activity observation. Raw BMI and calculated BMI data were then merged by using either the raw or calculated values when only one was available, and by taking the median when both were available.

# **Supplementary Results**

## Comparative analysis

Fitbit wear-time was correlated with overall activity as measured by Fitbit steps/day (r=-0.13, *P=*.001). Fitbit wear-time was not correlated with overall physical activity as measured by IPAQ MET-hrs/week (r=0.03, *P=*.43), nor was Fitbit wear-time correlated with BMI (r=0.06, *P=*.11).

# Supplementary References

1. Ipaq. Guidelines for Data Processing and Analysis of the International Physical Activity Questionnaire ( IPAQ ) – Short and Long Forms. Accessed July 12, 2017. www.ipaq.ki.se

# **Supplementary Figures**

Figure S1. Participant selection diagram for the two study samples.

*****


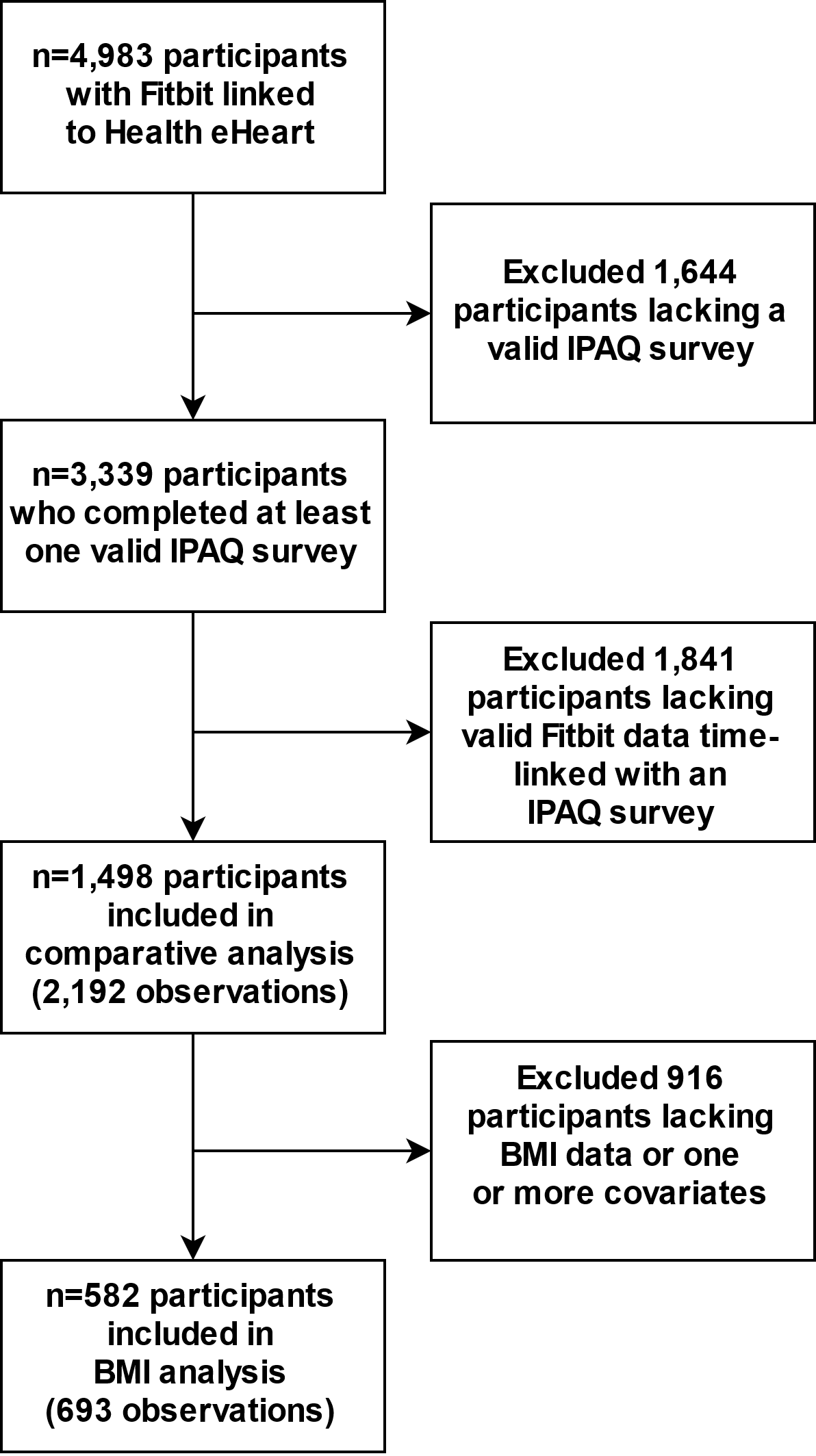


**586**

**697**

### Figure S2. Spearman rank correlations between all physical activity measurements acquired from Fitbits and self-report (IPAQ) for the 1,498 participants in the comparative analysis.


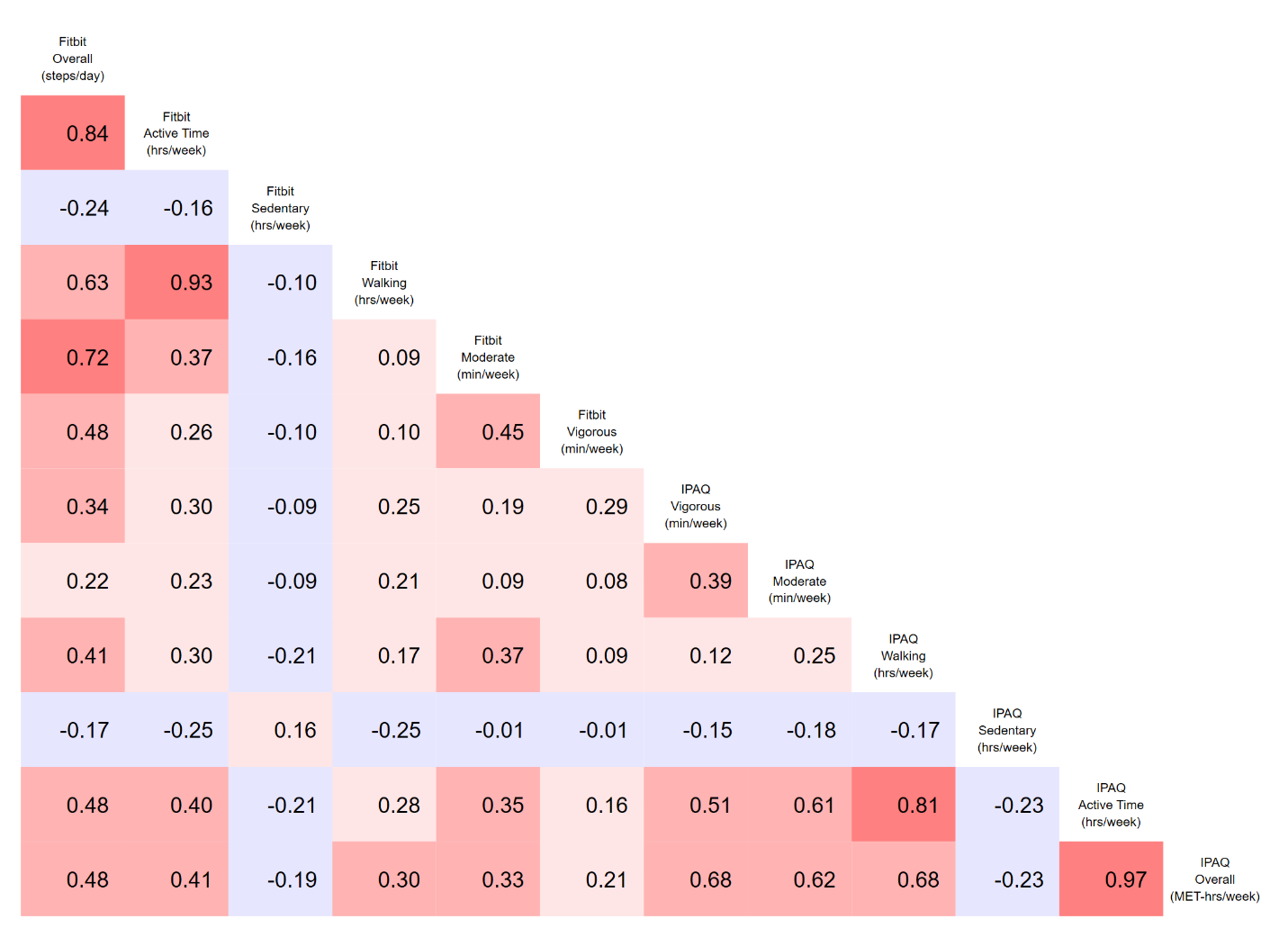


Matrix demonstrating Spearman rank correlations between all activity measurements acquired from Fitbits and IPAQs. The diagonal from bottom-left to center displays direct comparisons of equivalent measurements from Fitbits and IPAQs, which are also displayed as scatterplots in main text Fig. 1.

# Supplementary Tables

Table S1. **Fitbit devices used by 1,392/1,498 comparative analysis participants at the first activity observation.**

| Device | N | % | Cum. % | Wear Location |
| --- | --- | --- | --- | --- |
| Flex | 285 | 20.5 | (20.5) | Wrist |
| Charge HR | 263 | 18.9 | (39.4) | Wrist |
| One | 256 | 18.4 | (57.8) | Torso |
| Charge 2 | 161 | 11.6 | (69.3) | Wrist |
| MobileTrack | 81 | 5.8 | (75.1) | Torso |
| Charge | 63 | 4.5 | (79.7) | Wrist |
| Zip | 53 | 3.8 | (83.5) | Torso |
| Surge | 51 | 3.7 | (87.1) | Wrist |
| Flex 2 | 40 | 2.9 | (90.0) | Wrist |
| Blaze | 36 | 2.6 | (92.6) | Wrist |
| Alta | 35 | 2.5 | (95.1) | Wrist |
| Alta HR | 33 | 2.4 | (97.5) | Wrist |
| Ionic | 11 | 0.8 | (98.3) | Wrist |
| Force | 11 | 0.8 | (99.1) | Wrist |
| Ultra | 7 | 0.5 | (99.6) | Torso |
| Versa | 6 | 0.4 | (100.0) | Wrist |

Participants could link multiple devices over time; these devices were those that recorded data closest in time to and within 365 days of the first completed IPAQ. This data was not available for every participant.

Table S2. **Fitbit devices used by 586 BMI analysis participants at the first physical activity observation.**

| Device | N | % | Cum. % | Wear Location |
| --- | --- | --- | --- | --- |
| Flex | 130 | 22.2 | (22.2) | Wrist |
| Charge HR | 107 | 18.3 | (40.4) | Wrist |
| One | 105 | 17.9 | (58.4) | Torso |
| Charge 2 | 63 | 10.8 | (69.1) | Wrist |
| MobileTrack | 32 | 5.5 | (74.6) | Torso |
| Charge | 28 | 4.8 | (79.4) | Wrist |
| Zip | 26 | 4.4 | (83.8) | Torso |
| Surge | 22 | 3.8 | (87.5) | Wrist |
| Blaze | 16 | 2.7 | (90.3) | Wrist |
| Flex 2 | 15 | 2.6 | (92.8) | Wrist |
| Alta | 12 | 2.1 | (94.9) | Wrist |
| Alta HR | 11 | 1.9 | (96.8) | Wrist |
| Force | 9 | 1.5 | (98.3) | Wrist |
| Ionic | 5 | 0.9 | (99.2) | Wrist |
| Ultra | 3 | 0.5 | (99.7) | Torso |
| Versa | 2 | 0.3 | (100.0) | Wrist |

Participants could link multiple devices over time; these devices were those that recorded data closest in time to and within 365 days of the first completed IPAQ.

Table S3. **Full results for cluster robust regressions of BMI on overall physical activity measured by either Fitbit or self-report (IPAQ) in separate activity predictor models for 697 observations from 586 BMI analysis participants.**

| **BMI difference in kg/m^2^ per predictor (standard error, SE)** | | | | | | | | | |
| --- | --- | --- | --- | --- | --- | --- | --- | --- | --- |
|  | **Model 1** | **Model 2** | **Model 3** | **Model 4** |  | **Model 5** | **Model 6** | **Model 7** | **Model 8** |
| **Predictor Variable** | **β**  **(SE)** | **β**  **(SE)** | **β**  **(SE)** | **β**  **(SE)** | **Predictor Variable** | **β**  **(SE)** | **β**  **(SE)** | **β**  **(SE)** | **β**  **(SE)** |
| Fitbit Overall Activity (standardized) | -1.65*** (0.25) | -1.51*** (0.25) | -1.45*** (0.24) | -1.37*** (0.24) | IPAQ Overall Activity (standardized) | -0.79** (0.23) | -0.82*** (0.22) | -0.80*** (0.22) | -0.72** (0.22) |
| Wear time (hrs/day, mean-centered) | 0.40** (0.14) | 0.35* (0.14) | 0.35* (0.15) | 0.37* (0.14) | Wear time (hrs/day, mean-centered) | 0.30* (0.14) | 0.25 (0.15) | 0.26 (0.15) | 0.28* (0.14) |
| Data Collection Season (vs. Spring) |  |  |  |  | Data Collection Season (vs. Spring) |  |  |  |  |
| Summer | 1.06 (0.55) | 1.07* (0.54) | 1.10* (0.54) | 1.09* (0.53) | Summer | 1.16* (0.56) | 1.15* (0.56) | 1.19* (0.55) | 1.17* (0.54) |
| Fall | 0.39 (0.54) | 0.29 (0.52) | 0.20 (0.52) | 0.21 (0.51) | Fall | 0.50 (0.56) | 0.33 (0.54) | 0.21 (0.54) | 0.22 (0.53) |
| Winter | 0.35 (0.60) | 0.34 (0.59) | 0.35 (0.59) | 0.51 (0.57) | Winter | 0.38 (0.60) | 0.36 (0.59) | 0.36 (0.59) | 0.54 (0.57) |
| Device wear location (vs. wrist) |  |  |  |  | Device wear location (vs. wrist) |  |  |  |  |
| Torso | 0.79 (0.52) | 0.57 (0.52) | 0.69 (0.51) | 0.72 (0.51) | Torso | 0.53 (0.53) | 0.27 (0.53) | 0.42 (0.51) | 0.48 (0.51) |
| *Demographics* |  |  |  |  | *Demographics* |  |  |  |  |
| Age (years, mean-centered) |  | 0.02 (0.02) | 0.02 (0.02) | -0.01 (0.02) | Age (years, mean-centered) |  | 0.04* (0.02) | 0.04* (0.02) | 0.01 (0.02) |
| Male gender (vs. female) |  | -0.21 (0.50) | 0.03 (0.50) | -0.24 (0.49) | Male gender (vs. female) |  | -0.18 (0.50) | 0.08 (0.51) | -0.23 (0.50) |
| Education (vs. post-graduate degree) |  |  |  |  | Education (vs. post-graduate degree) |  |  |  |  |
| Bachelor’s degree |  | 0.75 (0.63) | 0.80 (0.63) | 0.85 (0.62) | Bachelor’s degree |  | 1.05 (0.64) | 1.08 (0.63) | 1.12  (0.62) |
| Less than Bachelor's |  | 1.58* (0.63) | 1.56* (0.64) | 1.40* (0.61) | Less than Bachelor's |  | 1.76** (0.66) | 1.72** (0.66) | 1.53** (0.63) |
| Income (annual thousands, vs. greater than $100) |  |  |  |  | Income (annual thousands, vs. greater than $100) |  |  |  |  |
| $50 to 100 |  | 1.18 (0.61) | 1.05 (0.60) | 0.75 (0.59) | $50 to 100 |  | 1.33* (0.61) | 1.16 (0.60) | 0.84 (0.59) |
| Less than $50 |  | 1.31 (0.78) | 1.08 (0.79) | 0.59 (0.82) | Less than $50 |  | 1.71* (0.82) | 1.40 (0.84) | 0.87 (0.87) |

- Table S3 continued on the next page –

- Table S3 continued -

| **BMI difference in kg/m^2^ per predictor (standard error, SE)** | | | | | | | | | |
| --- | --- | --- | --- | --- | --- | --- | --- | --- | --- |
|  | **Model 1** | **Model 2** | **Model 3** | **Model 4** |  | **Model 5** | **Model 6** | **Model 7** | **Model 8** |
| **Predictor Variable** | **β**  **(SE)** | **β**  **(SE)** | **β**  **(SE)** | **β**  **(SE)** | **Predictor Variable** | **β**  **(SE)** | **β**  **(SE)** | **β**  **(SE)** | **β**  **(SE)** |
| Race (vs. White) |  |  |  |  | Race (vs. White) |  |  |  |  |
| Black/African-American |  | 2.08* (1.05) | 2.09* (1.01) | 2.17* (0.98) | Black/African-American |  | 2.07 (1.11) | 2.11* (1.07) | 2.18* (1.05) |
| Asian |  | -2.05* (0.87) | -2.23** (0.89) | -2.17** (0.78) | Asian |  | -2.24* (0.95) | -2.57** (0.96) | -2.39** (0.84) |
| Multiracial |  | -0.96 (1.21) | -0.87 (1.23) | -0.84 (1.16) | Multiracial |  | -1.01 (1.27) | -0.91 (1.28) | -0.85 (1.19) |
| Other |  | 2.53 (2.63) | 2.10 (2.54) | 1.55 (2.37) | Other |  | 2.22  (2.90) | 1.72 (2.77) | 1.20 (2.56) |
| Hispanic/Latino Ethnicity |  | 1.50 (1.44) | 1.53 (1.44) | 1.59 (1.44) | Hispanic/Latino Ethnicity |  | 1.50 (1.53) | 1.56 (1.51) | 1.60 (1.50) |
| *Health-Related Behaviors* |  |  |  |  | *Health-Related Behaviors* |  |  |  |  |
| Smoking History (vs. never) |  |  |  |  | Smoking History (vs. never) |  |  |  |  |
| Past |  |  | -0.07 (0.52) | -0.06 (0.50) | Past |  |  | 0.01 (0.52) | 0.01 (0.51) |
| Current |  |  | 2.06 (1.51) | 1.99 (1.44) | Current |  |  | 2.66 (1.54) | 2.56 (1.47) |
| Alcohol (drinks/week,  mean-centered) |  |  | -0.10** (0.03) | -0.09* (0.03) | Alcohol (drinks/week,  mean-centered) |  |  | -0.11** (0.03) | -0.10** (0.04) |
| *Clinical Characteristics* |  |  |  |  | *Clinical Characteristics* |  |  |  |  |
| Coronary artery disease |  |  |  | -2.50** (0.87) | Coronary artery disease |  |  |  | -2.35** (0.90) |
| Diabetes |  |  |  | 0.73 (0.75) | Diabetes |  |  |  | 0.71 (0.76) |
| Hyperlipidemia |  |  |  | 0.28 (0.53) | Hyperlipidemia |  |  |  | 0.33 (0.54) |
| Hypertension |  |  |  | 2.75*** (0.71) | Hypertension |  |  |  | 2.87*** (0.71) |
| *Model Characteristics* |  |  |  |  | *Model Characteristics* |  |  |  |  |
| Constant | 27.1*** (0.44) | 25.9*** (0.60) | 25.8*** (0.63) | 25.1*** (0.62) | Constant | 27.1*** (0.45) | 25.7*** (0.59) | 25.6*** (0.62) | 24.8*** (0.61) |
| R-squared | 0.087 | 0.136 | 0.150 | 0.195 | R-squared | 0.030 | 0.094 | 0.112 | 0.160 |
| Degrees of freedom | 585 | 585 | 585 | 585 | Degrees of freedom | 585 | 585 | 585 | 585 |
| BIC | 4455 | 4489 | 4498 | 4485 | BIC | 4498 | 4522 | 4527 | 4514 |

One SD for overall activity was 3,663 steps/day by Fitbit and 37.2 MET-hrs/week by IPAQ. Continuous variables were centered and categorical variables referenced against the most common value. The constant in models 4 and 8 estimate BMI for a representative study participant: a 52 year-old, white, not Hispanic/Latino woman with a post-graduate degree and income in excess of $100,000 annually, who has never smoked regularly and has on average 5 standard drinks per week, and who denies coronary artery disease, diabetes, hyperlipidemia, or hypertension, who used a wrist-worn Fitbit for 17.2 hrs/day that counted an average of 8,778 daily steps during the seven days in which she self-reported 44.7 MET-hrs/week energy expenditure on an IPAQ completed online in Spring.

** P*<.05 for cluster-corrected association with BMI (582 participant clusters)

** *P*<.01 for cluster-corrected association with BMI (582 participant clusters)

*** *P*<.001 for cluster-corrected association with BMI (582 participant clusters)

Table S4. **Cluster robust regressions of BMI on Fitbit-measured and self-reported (IPAQ) overall activity in natural units across 697 observations from 586 participants.**

|  | **BMI difference (kg/m^2^) per predictor increase (95% CI)** | | | | | | | | |
| --- | --- | --- | --- | --- | --- | --- | --- | --- | --- |
| **Activity Predictor** | **Unadjusted** | | **Adjusted for Demographics** | | **Adjusted for Demographics and Health-Related Behaviors** | | **Adjusted for Demographics, Health-Related Behaviors, and Clinical Characteristics** | | |
| Overall Activity, Fitbit per 3,000 steps/day and IPAQ per 20 MET-hrs/week increase | | | | | | | | | |
| *Separate Predictor Models* | | | | | | | | | |
| Fitbit | -1.39*** | (-1.81 - -0.97) | -1.27*** | (-1.67 - -0.87) | -1.22*** | (-1.62 - -0.82) | -1.15*** | (-1.54 - -0.76) |  |
| IPAQ | -0.43*** | (-0.67 - -0.19) | -0.44*** | (-0.68 - -0.21) | -0.43*** | (-0.67 - -0.20) | -0.39** | (-0.62 - -0.16) |  |
| *Combined Predictor Model* | | | | | | | | | |
| Fitbit | -1.32*** | (-1.76 - -0.88) | -1.17*** | (-1.60 - -0.75) | -1.12*** | (-1.54 - -0.70) | -1.07*** | (-1.50 - -0.65) |  |
| IPAQ | -0.13 | (-0.36 - 0.10) | -0.16 | (-0.39 - 0.07) | -0.17 | (-0.40 - 0.07) | -0.14 | (-0.38 - 0.10) |  |

Cluster robust regressions with 697 activity observations from 586 participants. Overall activity units were set heuristically (Fitbit, per 3,000 steps/day increase; IPAQ, per 20 MET-hrs/week increase). Fitbit and IPAQ measured the same seven days. All models were adjusted for Fitbit wear-time, data collection season, and Fitbit device wear location. Demographics include age, gender, education, income, race, and ethnicity. Health-related behaviors include smoking and alcohol use. Clinical characteristics include coronary artery disease, diabetes, hyperlipidemia, and hypertension.

** P*<.05 for cluster-corrected association with BMI (586 participant clusters)

** *P*<.01 for cluster-corrected association with BMI (586 participant clusters)

*** *P*<.001 for cluster-corrected association with BMI (586 participant clusters)

Table S5. **Cluster robust regressions of BMI on standardized Fitbit-measured and self-reported (IPAQ) activity by intensity across 697 observations from 586 participants.**

|  | **BMI difference (kg/m^2^) per SD predictor increase (95% CI)** | | | | | | | | |
| --- | --- | --- | --- | --- | --- | --- | --- | --- | --- |
| **Activity Predictor** | **Unadjusted** | | **Adjusted for Demographics** | | **Adjusted for Demographics and Health-Related Behaviors** | | **Adjusted for Demographics, Health-Related Behaviors, and Clinical Characteristics** | | |
| **Active Time^a^** | | | | | | | | | |
| *Separate Predictor Models* | | | | | | | | | |
| Fitbit | -1.65*** | (-2.13 - -1.14) | -1.56*** | (-2.04 - -1.09) | -1.50*** | (-1.97 - -1.02) | -1.42*** | (-1.87 - -0.96) |  |
| IPAQ | -0.80*** | (-1.25 - -0.36) | -0.84*** | (-1.27 - -0.42) | -0.82*** | (-1.25 - -0.40) | -0.74*** | (-1.16 - -0.32) |  |
| *P* Value^b^ | .002 | | .004 | | .009 | | .01 | |  |
| *Combined Predictor Model* | | | | | | | | | |
| Fitbit | -1.52*** | (-2.03 - -1.01) | -1.43*** | (-1.92 - -0.93) | -1.36*** | (-1.86 - -0.86) | -1.30*** | (-1.79 - -0.82) |  |
| IPAQ | -0.34 | (-0.79 - 0.11) | -0.38 | (-0.81 - 0.06) | -0.38 | (-0.82 - 0.07) | -0.32 | (-0.77 - 0.12) |  |
| *P* Value^b^ | .003 | | .006 | | .01 | | .01 | |  |
| **Sedentary Time^c^** | | | | | | | | | |
| *Separate Predictor Models* | | | | | | | | | |
| Fitbit | 0.94** | (0.25 - 1.64) | 0.93** | (0.28 - 1.59) | 0.93** | (0.28 - 1.58) | 0.89** | (0.26 - 1.53) |  |
| IPAQ | 0.94*** | (0.45 - 1.44) | 1.10*** | (0.62 - 1.58) | 1.09*** | (0.61 - 1.58) | 1.03*** | (0.56 - 1.50) |  |
| *P* Value^b^ | .99 | | .64 | | .65 | | .69 | |  |
| *Combined Predictor Model* | | | | | | | | | |
| Fitbit | 0.72* | (0.04 - 1.39) | 0.63 | (-0.02 - 1.29) | 0.63 | (-0.02 - 1.29) | 0.61 | (-0.03 - 1.26) |  |
| IPAQ | 0.84** | (0.36 - 1.32) | 1.00*** | (0.52 - 1.48) | 0.99*** | (0.50 - 1.48) | 0.93*** | (0.47 - 1.40) |  |
| *P* Value^b^ | .77 | | .40 | | .42 | | .46 | |  |
| **Walking^d^** | | | | | | | | | |
| *Separate Predictor Models* | | | | | | | | | |
| Fitbit | -1.36*** | (-1.86 - -0.86) | -1.32*** | (-1.81 - -0.83) | -1.26*** | (-1.74 - -0.77) | -1.19*** | (-1.65 - -0.73) |  |
| IPAQ | -0.76** | (-1.21 - -0.32) | -0.78*** | (-1.20 - -0.35) | -0.73** | (-1.15 - -0.31) | -0.64** | (-1.06 - -0.22) |  |
| *P* Value^b^ | .049 | | .06 | | .08 | | .06 | |  |
| *Combined Predictor Model* | | | | | | | | | |
| Fitbit | -1.24*** | (-1.75 - -0.74) | -1.20*** | (-1.69 - -0.70) | -1.14*** | (-1.64 - -0.65) | -1.09*** | (-1.57 - -0.62) |  |
| IPAQ | -0.53* | (-0.98 - -0.07) | -0.54* | (-0.97 - -0.10) | -0.51* | (-0.95 - -0.07) | -0.43 | (-0.87 - 0.01) |  |
| *P* Value^b^ | .06 | | .08 | | .09 | | .08 | |  |

- Table S5 continued on next page -

- Table S5 continued -

|  | **BMI difference (kg/m^2^) per SD predictor increase (95% CI)** | | | | | | | |
| --- | --- | --- | --- | --- | --- | --- | --- | --- |
| **Activity Predictor** | **Unadjusted** | | **Adjusted for Demographics** | | **Adjusted for Demographics and Health-Related Behaviors** | | **Adjusted for Demographics, Health-Related Behaviors, and Clinical Characteristics** | |
| **Moderate Activity^e^** | | | | | | | | |
| *Separate Predictor Models* | | | | | | | | |
| Fitbit | -1.01*** | (-1.57 - -0.44) | -0.88** | (-1.42 - -0.34) | -0.85** | (-1.38 - -0.32) | -0.83** | (-1.36 - -0.29) |
| IPAQ | -0.39 | (-0.83 - 0.05) | -0.47* | (-0.91 - -0.02) | -0.50* | (-0.92 - -0.07) | -0.49* | (-0.90 - -0.07) |
| *P* Value^b^ | .10 | | .26 | | .32 | | .34 | |
| *Combined Predictor Model* | | | | | | | | |
| Fitbit | -0.98** | (-1.55 - -0.42) | -0.85** | (-1.39 - -0.30) | -0.81** | (-1.34 - -0.28) | -0.79** | (-1.33 - -0.25) |
| IPAQ | -0.31 | (-0.74 - 0.12) | -0.39 | (-0.83 - 0.05) | -0.42 | (-0.84 - 0.00) | -0.41 | (-0.83 - 0.00) |
| *P* Value^b^ | .10 | | .26 | | .32 | | .34 | |
| **Vigorous Activity^f^** | | | | | | | | |
| *Separate Predictor Models* | | | | | | | | |
| Fitbit | -0.85*** | (-1.22 - -0.49) | -0.75*** | (-1.09 - -0.40) | -0.73*** | (-1.07 - -0.39) | -0.60** | (-0.97 - -0.23) |
| IPAQ | -0.52* | (-0.92 - -0.12) | -0.50* | (-0.89 - -0.11) | -0.49* | (-0.89 - -0.09) | -0.42* | (-0.83 - -0.01) |
| *P* Value^b^ | .18 | | .30 | | .31 | | .48 | |
| *Combined Predictor Model* | | | | | | | | |
| Fitbit | -0.79*** | (-1.15 - -0.43) | -0.68*** | (-1.02 - -0.34) | -0.67*** | (-1.00 - -0.33) | -0.55** | (-0.91 - -0.18) |
| IPAQ | -0.39 | (-0.79 - 0.00) | -0.38 | (-0.77 - 0.00) | -0.38 | (-0.77 - 0.02) | -0.33 | (-0.74 - 0.08) |
| *P* Value^b^ | .18 | | .28 | | .31 | | .47 | |

Cluster robust regressions on 697 activity observations from 586 participants. Fitbit and IPAQ measured the same seven days. All models were adjusted for Fitbit wear-time, data collection season, and Fitbit device wear location. Demographics include age, gender, education, income, race, and ethnicity. Health-related behaviors include smoking and alcohol use. Clinical characteristics include coronary artery disease, diabetes, hyperlipidemia, and hypertension.

^a^ 1 SD for active time was 7.3 hrs/week for Fitbit and 8.4 hrs/week for IPAQ

^b^ *P* values for Wald tests comparing Fitbit vs. self-report regression coefficients

^c^ 1 SD for sedentary time was 10.7 hrs/week for Fitbit and 24.5 hrs/week for IPAQ

^d^ 1 SD for walking time was 6.9 hrs/week for Fitbit and 5.3 hrs/week for IPAQ

^e^ 1 SD for moderate activity was 122 min/week for Fitbit and 222 min/week for IPAQ

^f^ 1 SD for vigorous activity was 45 min/week for Fitbit and 147 min/week for IPAQ

** P*<.05 for cluster-corrected association with BMI (586 participant clusters)

** *P*<.01 for cluster-corrected association with BMI (586 participant clusters)

*** *P*<.001 for cluster-corrected association with BMI (586 participant clusters)
